# Angstrom-Scale Active Width Control of Nano Slits for Variable Plasmonic Cavity

**DOI:** 10.3390/nano11092463

**Published:** 2021-09-21

**Authors:** Dukhyung Lee, Dohee Lee, Hyeong Seok Yun, Dai-Sik Kim

**Affiliations:** Department of Physics and Center for Atom Scale Electromagnetism, Ulsan National Institute of Science and Technology, Ulsan 44919, Korea; dodong13@unist.ac.kr (D.L.); yoonhs1432@unist.ac.kr (H.S.Y.)

**Keywords:** nanogap, flexible substrate, plasmonic cavity, active control, displacement ratio, angstrom-scale

## Abstract

Nanogap slits can operate as a plasmonic Fabry–Perot cavity in the visible and infrared ranges due to the gap plasmon with an increased wavenumber. Although the properties of gap plasmon are highly dependent on the gap width, active width tuning of the plasmonic cavity over the wafer length scale was barely realized. Recently, the fabrication of nanogap slits on a flexible substrate was demonstrated to show that the width can be adjusted by bending the flexible substrate. In this work, by conducting finite element method (FEM) simulation, we investigated the structural deformation of nanogap slit arrays on an outer bent polydimethylsiloxane (PDMS) substrate and the change of the optical properties. We found that the tensile deformation is concentrated in the vicinity of the gap bottom to widen the gap width proportionally to the substrate curvature. The width widening leads to resonance blueshift and field enhancement decrease. Displacement ratio ((width change)/(supporting stage translation)), which was identified to be proportional to the substrate thickness and slit period, is on the order of 10^−5^ enabling angstrom-scale width control. This low displacement ratio comparable to a mechanically controllable break junction highlights the great potential of nanogap slit structures on a flexible substrate, particularly in quantum plasmonics.

## 1. Introduction

Metal gaps with a width of a few nanometers have attracted much attention in nanophotonics because of gap plasmon generation accompanied by extreme light confinement and field enhancement [1,2,3,4]. Since gap plasmon wavelength is compressed compared to the free space wavelength, a Fabry–Perot cavity for the visible and infrared ranges is established along the thickness direction inside a nanogap slits. Using nanogap slits as a plasmonic cavity, several studies have reported exciting phenomena including enhanced light-matter interaction and resonant transmission [5,6,7,8]. In the applications, precise gap width control is a pivotal factor because the resonance properties such as the gap plasmon wavenumber are determined by the gap width. The importance of width control is much more emphasized in the quantum plasmonic regime where tunneling current across a nanogap is exponentially dependent on the barrier width [9,10,11,12,13].

Atomic layer lithography which combines photolithography and atomic layer deposition was developed a few years ago for high-throughput wafer-scale fabrication of precisely width-defined nanogap structures [1,14,15,16]. Using this technique, we can define a nanogap slit width as the thickness of a dielectric spacer grown by atomic layer deposition with an atomic precision uniformly over the whole slit length. While electron- or ion-beam lithography suffers from limited aspect ratios (feature width/feature depth) [17], atomic layer lithography defines the nanogap width independently of the thickness without limitation on aspect ratio. However, atomic-scale active width control on a nanogap slit has been barely developed, which is partially due to the difficulty of uniform operation over the whole length. For other plasmonic or Mie structures, several studies have demonstrated active structural control exploiting various methods [18,19,20,21,22,23]. Especially, nanometer-scale interparticle distance control was achieved for plasmonic nanoparticles on a flexible PDMS substrate by bending or stretching the substrate [20,21,22]. Recently, atomic layer lithography was successfully applied to the fabrication of nanogap slits on a flexible PET substrate to demonstrate that the nanogap width can be actively controlled by bending the substrate [24,25]. Considering the importance in applications, it is highly anticipated to further investigate the mechanical and optical aspects of active width control of nanogap slits on a flexible substrate.

In this work, we conducted a simulation study on active gap width control using a sliver nanogap slit array on a PDMS substrate as a model system. It was demonstrated that noble metal patterns on a PDMS substrate can be successfully fabricated with good adhesion if proper surface treatments are employed [26,27]. In outer bending situations, we examined the gap width increase and the corresponding changes of the plasmonic cavity. Investigation on the geometrical factors reveals that the gap width can be controlled with angstrom-scale due to the low displacement ratio, which is especially valuable in quantum plasmonic applications. Because this substrate bending strategy can be applied to a well-defined slit cavity fabricated by atomic layer lithography to enable precise active control of the plasmonic resonance, it will be useful for various plasmonic applications including surfaced enhanced Raman spectroscopy, strain sensing and active color filter [20,21,22,23,24].

## 2. Materials and Methods

**Figure 1:**
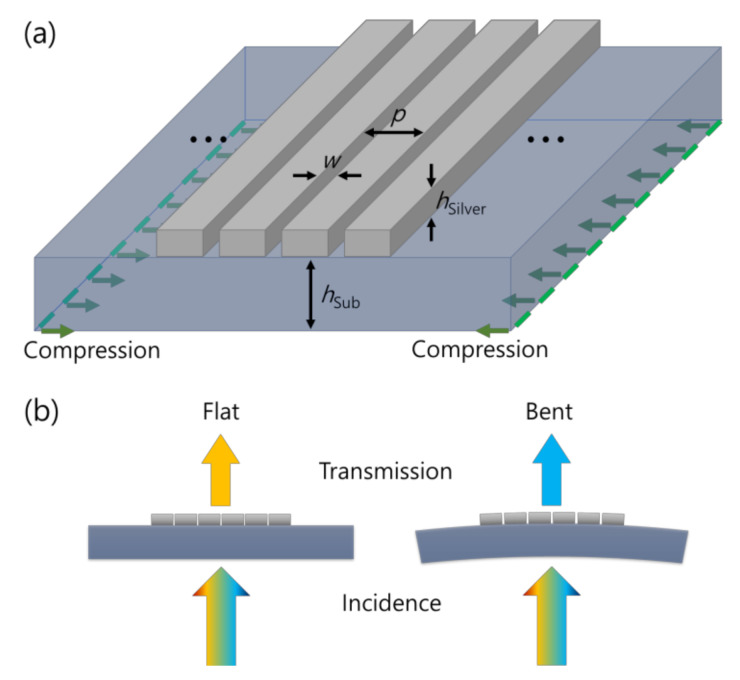
Concept of the nanogap slit system on a PDMS substrate. (**a**) A schematic of the slit array geometry denoting some important geometrical factors. *w*_0_ = 3 nm, *p* = 500 nm, *h*_Silver_ = 200 nm and *h*_Sub_ = 250 μm. The green arrows denote direction of the displacement applied in the structural mechanic’s simulation for outer bending. (**b**) An operation schematic of the nanogap slit as a variable plasmonic cavity.

A schematic of the nanogap system is illustrated in Figure 1a. We generated an outer bending situation by applying inward displacement at the two bottom edges of the PDMS substrate. After obtaining the geometrical deformation, the corresponding optical changes were examined with a plane wave illumination from the substrate side assuming a cavity application illustrated in Figure 1b.

We used commercial software for the finite element method simulation (COMSOL Multiphysics 5.6, structural mechanics and wave optics modules). The initial width, period, and thickness of the silver nanogap slits were set to be *w*_0_ = 3 nm, *p* = 500 nm and *h*_Silver_ = 200 nm and the PDMS thickness was *h*_Sub_ = 250 μm. Young’s modulus, Poisson’s ratio and refractive index of the PDMS substrate was set to be 1.32 MPa, 0.499 [28] and 1.38 [29]. Silver was assumed to have Young’s modulus of 81.1GPa and Poisson’s ratio of 0.368. The tabulated refractive index given by Babar and Weaver for silver was used [30]. All the simulations were conducted in 2D geometry, justified by the actual slit lengths of 20 μm to 2 cm of real samples [24,25]. For the structural mechanic’s simulation, assuming a real dimension of the substrate as 1 mm by 1 mm, we exploited generalized plane strain condition which is suitable for geometries of no external out-of-plane stress [31]. The curvature of a bent substrate at the center was obtained by fitting the substrate middle line within the ±0.5 mm range to a quadratic. The number of the slits is 40 and we evaluated gap width and periodicity for the center slit. For the optics simulation, we applied a periodic condition with the changed gap width and period. The spectral range of vacuum wavelengths of 1–2 μm was investigated in a step size of 5 nm.

## 3. Results

**Figure 2:**
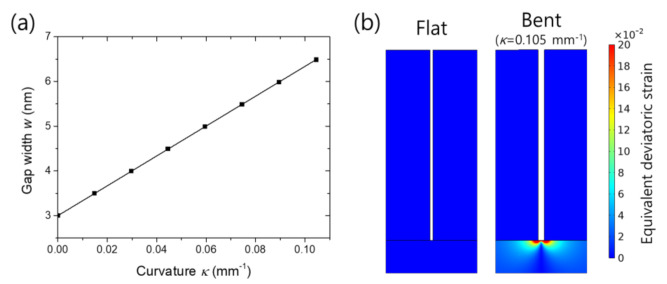
Nanogap slit deformation during bending: (**a**) Gap width versus substrate curvature with a linear fit (solid line); (**b**) Distributions of equivalent deviatoric strain in the vicinity of a slit for the flat and bent (*κ* = 0.105 mm^−1^) cases.

The slit width was found to increase proportionally to the substrate curvature as indicated by the good linear fit in Figure 2a (see Table S1 in Supplementary Materials for the fitting equation and residual standard deviation). When the curvature is 0.105 mm^−1^, the gap width extended to 6.48 nm, which is more than twice the initial width. Figure 2b displays distributions of equivalent deviatoric strain (*ε*_deve_ = ((2/3)dev(ε)_ij_∙dev(ε)_ji_)^1/2^ where dev(ε)_ij_ = ε_ij_ – (ε_kk_/3)Δ_ij_), or degree of deformation, for the flat and bent cases. In the bent state, tensile deformation is concentrated at the gap bottom, increasing the slit width. On the contrary, because silver is much stiffer than PDMS in terms of Young’s modulus, the silver parts are barely deformed as shown on the side walls of the slit that remain almost parallel. We note that the gap width difference between the top and the bottom given by the arc length equation *l* = *rθ* is negligible because the metal height is much smaller than the radius of curvature within the simulated range.

**Figure 3:**
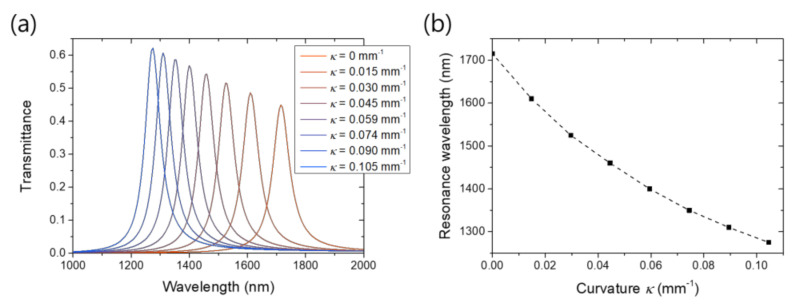
Blueshift of the Fabry–Perot resonance: (**a**) Transmission spectra for the various bending curvature; (**b**) Resonance wavelength as a function of the curvature.

An important consequence of the slit widening is the resonance blueshift of the plasmonic cavity. Figure 3a shows the transmission spectra for the various bending curvatures. The initial resonance is at the wavelength of 1715 nm. In a wider gap, because the gap plasmon for a given free-space wavelength has a smaller wavenumber, or a lower effective refractive index [32], the Fabry–Perot resonance condition for the fixed thickness is matched at a shorter free-space wavelength. For the curvature of 0.105 mm^−1^, the resonance wavelength becomes 1275 nm, which is three-quarters of the initial one. The resonance wavelength is plotted as a function of the curvature in Figure 3b. The gradual slowing down of the shift rate reflects the fact that the width dependence of gap plasmon is higher when the gap width is narrower, which again emphasizing the importance of precise width control for nanogaps.

**Figure 4:**
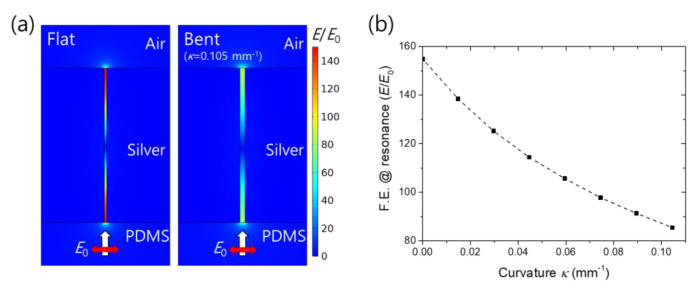
Field enhancement modulation: (**a**) Electric field distributions in the vicinity of a slit for the flat and bent (*κ* = 0.105 mm^−1^) cases at their respective resonances. The white and red arrows denote the light incident direction and polarization, respectively. The electric fields were normalized by the incident field *E*_0_; (**b**) Field enhancement factor (*E*/*E*_0_) at the resonance as a function of the curvature. Average values were taken at the top exit.

Another important consequence is the modulation of the field enhancement in the nanogap. At the Fabry–Perot resonance, electric field antinodes are established at the top and bottom exits of a slit. The enhanced electric field at the antinodes plays a crucial role in the applications of light-matter interaction. Although the antinodes are well established in both the flat and bent cases, the wider gap in the bent case results in the lower field enhancement, as shown in Figure 4a. Field enhancement averaged at the top exit at the resonance is displayed as a function of the curvature in Figure 4b, confirming the decreasing tendency. The modulation range is almost two-fold for the curvature range of 0 to 0.105 mm^−1^.

The above results demonstrate that a nanogap slit on a flexible substrate can be exploited as a mechanically variable plasmonic cavity. In the following section, we discuss geometrical factors determining the precision and dynamic range of the active control and implications on quantum plasmonics.

## 4. Discussion

**Figure 5:**
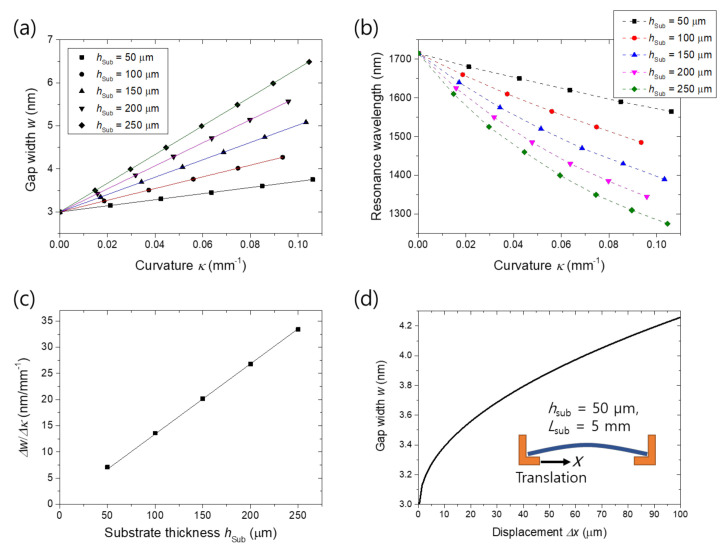
Effect of the substrate thickness on the active width control: (**a**) Gap width versus substrate curvature for PDMS substrate thicknesses *h*_sub_ of 50, 100, 150, 200 and 250 μm; (**b**) Resonance wavelength as a function of the curvature for the various PDMS thickness; (**c**) Δ*w*/Δ*κ*, the slope in (**a**) versus substrate thickness; (**d**) Gap width versus displacement of the supporting stage for the case of *h*_sub_ = 50 μm when the substrate length is 5 mm. Bending is assumed to be realized by translating a supporting stage as illustrated in the inset. Solid lines in (**a**,**c**) are linear fits.

We found that the gap width change rate is proportional to the substrate thickness. We conducted simulations by varying the substrate thickness, keeping the other parameters the same. As shown in Figure 5a,b, a thicker substrate results in a faster gap widening, and thus a faster resonance shift. Linear fitting to the width–curvature graphs in Figure 5a provides width change rate Δ*w*/Δ*κ* as a function of the substrate thickness. Width change rate Δ*w*/Δ*κ* in Figure 5c clearly reveals the proportionality to the substrate thickness, which is consistent with the expression *ε*_norm_ = *h*_Sub_∙*κ*/2 for the nominal strain of a bent substrate [33].

The rate Δ*w*/Δ*κ* is translated into displacement ratio, or ratio of gap width change to external stage movement if a specific experimental configuration is provided. We estimated the displacement ratio for the case of *h*_Sub_ = 50 μm assuming a typical experimental setup where external stress is applied by translating a supporting stage in the horizontal (*x*) direction as illustrated in the inset of Figure 5d. The substrate curvature at the center is given by

*κ* = (2π/*L*_sub_)∙((Δ*x*/*L*_sub_) − (*π*^2^*h*_sub_^2^/12*L*_sub_^2^))^1/2^ (1)

for a displacement Δ*x* and a substrate length *L*_sub_ [33]. Setting *L*_sub_ to 5 mm which is a typical device length, from Equation (1) and the obtained rate Δ*w*/Δ*κ*, gap width was calculated as a function of the stage displacement as shown in Figure 5d. The displacement ratio estimated from the curve in Figure 5d is on the order of 10^−5^. This low displacement ratio suggests that angstrom-scale width control can be achieved by micrometer-scale stage translation.

**Figure 6:**
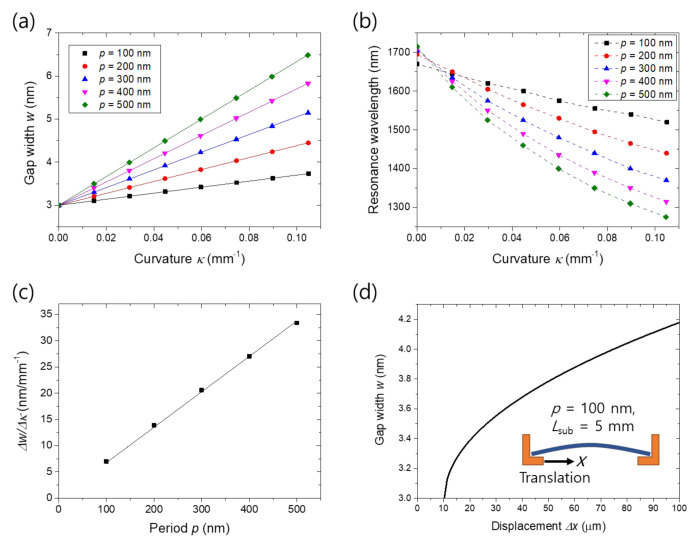
Effect of the slit period on the active width control: (**a**) Gap width versus substrate curvature for slit periods of 100, 200, 300, 400 and 500 nm; (**b**) Resonance wavelength as a function of the curvature for the various slit period; (**c**) Δ*w*/Δ*κ*, the slope in (**a**), versus slit period; (**d**) Gap width versus displacement of the supporting stage for the case of *p* = 100 nm when the substrate length is 5 mm. Solid lines in (**a**) and (**c**) are linear fits.

Slit periodicity was also found to be proportional to width change rate as summarized in Figure 6a,c. In the structural mechanic’s simulation, to maintain the overall lateral extent of the slit array, slit numbers were set to be 200, 100, 66, 50 and 40 for the periods of 100, 200, 300, 400 and 500 nm, respectively. For the other parameters, we used the values given in the Methods section. The proportionality is attributed to the fact that all the tensile deformation within one slit period is concentrated to the gap bottom. That is, the overall strain of the slit array is maintained independently of the period. Accordingly, the speed of resonance shift slows down as the period decreases as shown in Figure 6b. The slight differences between the initial resonance wavelengths are due to surface plasmon coupling between the slits. The width–displacement curve for the case of *p* = 100 nm obtained by the same method as Figure 5d is presented in Figure 6d, verifying the low displacement ratio of 10^−5^. The difference between the starting points in Figure 5d and Figure 6d is due to the difference in the critical strain *π*^2^*h*_sub_^2^/12*L*_sub_^2^ where the substrate starts to bend [33]. Effect of the geometrical factors given in Figure 5 and Figure 6 can be summarized into the expression:

Δ*w* = *α*∙*h*_Sub_∙*p*∙*κ*, (2)

where *α* is 0.267.

**Figure 7:**
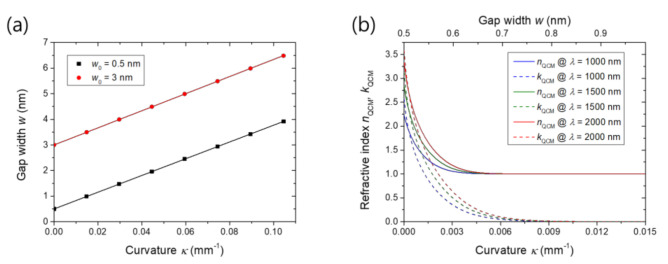
Effect of the initial gap width and quantum tunneling: (**a**) Gap width versus substrate curvature for initial gap widths of 0.5 and 3 nm. Solid lines are linear fits; (**b**) Real and imaginary parts of the refractive indexes *n*_QCM_, *k*_QCM_ of air in the gap calculated from the quantum corrected model for wavelengths of 1000, 1500 and 2000 nm. The initial gap width is 0.5 nm. The curvature range of 0 to 0.015 mm^−1^ on the bottom axis is translated into the gap width range of 0.5 to 0.99 nm on the top axis.

The initial gap width is expected to be irrelevant to the rate Δ*w*/Δ*κ* because the overall strain should be the same for the same substrate curvature. Indeed, Figure 7a verifies that the rate Δ*w*/Δ*κ* is the same for the initial widths of 0.5 and 3 nm, implying that the 0.5 nm gap also can be controlled in the angstrom scale. For the other parameters, we used the values given in the Methods section. Angstrom-scale width control becomes especially important for a gap whose width is narrow enough for electrons to flow across the gap via quantum tunneling: the 0.5 nm gap is the case. Tunneling current alters the dielectric environment inside the gap or makes the air a slightly conducting medium. Because tunneling current decreases exponentially as the gap width increases, only a few angstrom width changes in the quantum regime can significantly shift and modulate the plasmonic resonance.

To examine how much the active width control system provides tunability on the quantum plasmonic effect, we estimated the refractive index of the air inside the gap as a function of the substrate curvature for the case of the 0.5 nm initial width. For this estimation, we first obtained the relationship between the curvature and the gap width from the simulation given in Figure 7a. For a given gap width, the refractive index was estimated using the quantum corrected model suggested by Esteban et al. [13] where the gap medium is treated as a Drude metal. Tunneling damping *γ*_g_ in the quantum corrected model is given as

*γ*_g_ = ε_0_*ω*_g_^2^/*σ*_0_, (3)

where *ω*_g_ was assumed to be the plasma frequency of silver 13.7 PHz and *σ*_0_ is the static tunneling conductivity. We calculated the static tunneling conductivity *σ*_0_ using the Simmons equation, assuming a Fermi energy of 5.49 eV, a work function of 4.64 eV and an applied voltage of 0.1 μV. The low applied voltage ensures the exclusion of the voltage-related nonlinearity. Then, the quantum corrected model provides the local permittivity of the gap medium as

*ε* = 1 − *ω*_g_^2^/(*ω*^2^ + *iωγ*_g_). (4)

Taking the square root of Equation (4), we obtained the complex refractive index. The estimated real and imaginary parts of the refractive indexes are displayed in Figure 7b as solid and dotted lines, respectively, for wavelengths of 1000, 1500 and 2000 nm. The real and imaginary parts are in the range of 2 to 4 for the flat substrate and decrease with the curvature to 1 and 0, respectively, which are the refractive index of air in free space. Most of this refractive index modulation occurs in the range where the gap width is less than 0.8 nm or the curvature less than 0.009 mm^−1^, emphasizing the importance of the angstrom-scale control. Because the width is controlled uniformly at any height within the gap as shown in Figure 2b, unlike STM/AFM-based junctions and break junctions which are essentially for single point contact, the quantum plasmonic modulation effect presented in Figure 7b is applied over the whole slit structure.

## 5. Conclusions

We demonstrated that the nanogap slit width, and thus the optical properties of the plasmonic cavity can be controlled actively by applying mechanical bending to the PDMS substrate. Outer bending results in gap widening, resonance blueshift and field enhancement decrease. The width change rate is proportional to the substrate thickness and the slit period while independent of the initial width. The low displacement ratio on the order of 10^−5^, which is comparable to a mechanically controllable break junction [34], implies angstrom-scale width control and high mechanical stability. The nanogap system in this study is expected to be particularly useful in the field of quantum plasmonics because tunneling conductivity and the dielectric environment inside the gap can be controlled uniformly within the whole slit volume, overcoming the limit of the conventional junction-based techniques.

## Data Availability

The data presented in this study are available on request from the corresponding author.

## Supplementary Materials

The following are available online at https://www.mdpi.com/article/10.3390/nano11092463/s1, Table S1: Fitting equations and residual standard deviations in Figure 2a, Figure 5a,c, Figure 6a,c and Figure 7a.

## References

[1] Chen X., Park H.-R., Pelton M., Piao X., Lindquist N.C., Im H., Kim Y.J., Ahn J.S., Ahn K.J., Park N. (2013). Atomic layer lithography of wafer-scale nanogap arrays for extreme confinement of electromagnetic waves. Nat. Commun..

[2] Kim M.-K., Sim H., Yoon S.J., Gong S.-H., Ahn C.W., Cho Y.-H., Lee Y.-H. (2015). Squeezing Photons into a Point-Like Space. Nano Lett..

[3] Pedano M.L., Li S., Schatz G.C., Mirkin C.A. (2010). Periodic Electric Field Enhancement Along Gold Rods with Nanogaps. Angew. Chem..

[4] Sturges T.J., Repän T., Downing C.A., Rockstuhl C., Stobińska M. (2020). Extreme renormalisations of dimer eigenmodes by strong light–matter coupling. New J. Phys..

[5] Chen X., Ciracì C., Smith D.R., Oh S.-H. (2015). Nanogap-Enhanced Infrared Spectroscopy with Template-Stripped Wafer-Scale Arrays of Buried Plasmonic Cavities. Nano Lett..

[6] Kang T., Rhie J., Park J., Bahk Y.-M., Ahn J.S., Jeon H., Kim D.-S. (2015). Resonance tuning of electric field enhancement of nanogaps. Appl. Phys. Express.

[7] Ahn J.S., Kang T., Singh D.K., Bahk Y.-M., Lee H., Choi S.B., Kim D.-S. (2015). Optical field enhancement of nanometer-sized gaps at near-infrared frequencies. Opt. Express.

[8] Yang H., Kim D.-S., Kim R.H.J.-Y., Ahn J.S., Kang T., Jeong J., Lee D. (2018). Magnetic Nature of Light Transmission through a 5-nm Gap. Sci. Rep..

[9] Kim J.-Y., Kang B.J., Park J., Bahk Y.-M., Kim W.T., Rhie J., Jeon H., Rotermund F., Kim D.-S. (2015). Terahertz Quantum Plasmonics of Nanoslot Antennas in Nonlinear Regime. Nano Lett..

[10] Kim J.-Y., Kang B.J., Bahk Y.-M., Kim Y.S., Park J., Kim W.T., Rhie J., Han S., Jeon H., Park C.-H. (2016). Tunnelling current-voltage characteristics of Angstrom gaps measured with terahertz time-domain spectroscopy. Sci. Rep..

[11] Kang T., Kim R.H.J.-Y., Choi G., Lee J., Park H., Jeon H., Park C.-H., Kim D.-S. (2018). Terahertz rectification in ring-shaped quantum barriers. Nat. Commun..

[12] Bahk Y.-M., Kang B.J., Kim Y.S., Kim J.-Y., Kim W.T., Kim T.Y., Kang T., Rhie J., Han S., Park C.-H. (2015). Electromagnetic Saturation of Angstrom-Sized Quantum Barriers at Terahertz Frequencies. Phys. Rev. Lett..

[13] Esteban R., Borisov A.G., Nordlander P., Aizpurua J. (2012). Bridging quantum and classical plasmonics with a quantum-corrected model. Nat. Commun..

[14] Rhie J., Lee D., Bahk Y.-M., Jeong J., Choi G., Lee Y., Kim S., Hong S., Kim D.-S. (2018). Control of optical nanometer gap shapes made via standard lithography using atomic layer deposition. J. Micro/Nanolith. MEMS MOEMS.

[15] Jeong J., Rhie J., Jeon W., Hwang C.S., Kim D.-S. (2015). High-throughput fabrication of infinitely long 10 nm slit arrays for terahertz applications. J. Infrared Millim. Terahertz.

[16] Kim N., In S., Lee D., Rhie J., Jeong J., Kim D.-S., Park N. (2018). Colossal Terahertz Field Enhancement Using Split-Ring Resonators with a Sub-10 nm Gap. ACS Photonics.

[17] Semple M., Hryciw A.C., Li P., Flaim E., Iyer A.K. (2021). Patterning of Complex, Nanometer-Scale Features in Wide-Area Gold Nanoplasmonic Structures Using Helium Focused Ion Beam Milling. ACS Appl. Mater. Interfaces.

[18] Zhang F., Feng S., Qiu K., Liu Z., Fan Y., Zhang W., Zhao Q., Zhou J. (2015). Mechanically stretchable and tunable metamaterial absorber. Appl. Phys. Lett..

[19] Xu J., Fan Y., Yang R., Fu Q., Zhang F. (2019). Realization of switchable EIT metamaterial by exploiting fluidity of liquid metal. Opt. Express.

[20] Liu W., Shen Y., Xiao G., She X., Wang J., Jin C. (2017). Mechanically tunable sub-10nm metal gap by stretching PDMS substrate. Nanotechnology.

[21] Kang M., Kim J.-J., Oh Y.-J., Park S.-G., Jeong K.-H. (2014). A Deformable Nanoplasmonic Membrane Reveals Universal Correlations Between Plasmon Resonance and Surface Enhanced Raman Scattering. Adv. Mater..

[22] Sannomiya T., Hafner C., Vörös J. (2009). Strain mapping with optically coupled plasmonic particles embedded in a flexible substrate. Opt. Lett..

[23] Mitomo H., Horie K., Matsuo Y., Niikura K., Tani T., Naya M., Ijiro K. (2016). Active Gap SERS for the Sensitive Detection of Biomacromolecules with Plasmonic Nanostructures on Hydrogels. Adv. Opt. Mater..

[24] Kim D., Yun H.S., Das B., Rhie J., Vasa P., Kim Y.-I., Choa S.-H., Park N., Lee D., Bahk Y.-M. (2021). Topology-Changing Broadband Metamaterials Enabled by Closable Nanotrenches. Nano Lett..

[25] Das B., Yun H.S., Park N., Jeong J., Kim D.-S. (2021). A Transformative Metasurface Based on Zerogap Embedded Template. Adv. Opt. Mater..

[26] Wu J., Wang R., Yu H., Li G., Xu K., Tien N.C., Roberts R.C., Li D. (2015). Inkjet-printed microelectrodes on PDMS as biosensors for functionalized microfluidic systems. Lab Chip.

[27] Byun I., Coleman A.W., Kim B. (2013). Transfer of thin Au films to polydimethylsiloxane (PDMS) with reliable bonding using (3-mercaptopropyl)trimethoxysilane (MPTMS) as a molecular adhesive. J. Micromech. Microeng..

[28] Johnston I.D., McCluskey D.K., Tan C.K.L., Tracey M.C. (2014). Mechanical characterization of bulk Sylgard 184 for microfluidics and microengineering. J. Micromech. Microeng..

[29] Zhang X., Qiu J., Li X., Zhao J., Liu L. (2020). Complex refractive indices measurements of polymers in visible and near-infrared bands. Appl. Opt..

[30] Babar S., Weaver J.H. (2015). Optical constants of Cu, Ag, and Au revisited. Appl. Opt..

[31] Kamal S.M., Dixit U.S., Roy A., Liu Q., Silberschmidt V.V. (2017). Comparison of plane-stress, generalized-plane-strain and 3D FEM elastic–plastic analyses of thick-walled cylinders subjected to radial thermal gradient. Int. J. Mech. Sci..

[32] Gordon R., Brolo A.G. (2005). Increased cut-off wavelength for a subwavelength hole in a real metal. Opt. Express.

[33] Park S.-I., Ahn J.-H., Feng X., Wang S., Huang Y., Rogers J.A. (2008). Theoretical and Experimental Studies of Bending of Inorganic Electronic Materials on Plastic Substrates. Adv. Funct. Mater..

[34] Huang C., Rudnev A.V., Hong W., Wandlowski T. (2015). Break junction under electrochemical gating: Testbed for single-molecule electronics. Chem. Soc. Rev..

