# Supplementary material for: Angstrom-Scale Active Width Control of Nano Slits for Variable Plasmonic Cavity
Source: Nanomaterials (Basel). 2021 Sep 21;11(9):2463. doi: 10.3390/nano11092463 (PMC8465792; doi:10.3390/nano11092463)
Supplement: Supplementary file 1 [file nanomaterials-11-02463-s001.zip › nanomaterials-1367082-supplementary.pdf]

Supplementary Materials for

Angstrom-scale Active Width Control of Nano Slits for Variable Plasmonic Cavity

Dukhyung Lee *, Dohee Lee, Hyeong Seok Yun and Dai-Sik Kim *

Department of Physics and Center for Atom Scale Electromagnetism, Ulsan National Institute of Science and Technology, Ulsan, 44919, Republic of Korea

Correspondence: hyung0624@unist.ac.kr (Du.L.); daisikkim@unist.ac.kr (D.-S.K.)

Table S1. Fitting equations and residual standard deviations in Figures 2(a), 5(a), 5(c), 6(a), 6(c) and 7(a)

Item	Fitting equation	Obtained slope, α	Residual standard deviation
Figure 2(a)	$y = \alpha \cdot x + 3$	33.38974	0.00481
Figure 5(a), h_{Sub} = 50 μm	$y = \alpha \cdot x + 3$	7.09515	2.18695E-4
Figure 5(a), h_{Sub} = 100 μm	$y = \alpha \cdot x + 3$	13.59455	6.73019E-4
Figure 5(a), h_{Sub} = 150 μm	$y = \alpha \cdot x + 3$	20.19799	0.00177
Figure 5(a), h_{Sub} = 200 μm	$y = \alpha \cdot x + 3$	26.80505	0.00267
Figure 5(a), h_{Sub} = 250 μm	$y = \alpha \cdot x + 3$	33.38974	0.00481
Figure 5(c)	$y = \alpha \cdot x$	0.1342	0.22931
Figure 6(a), p = 100 nm	$y = \alpha \cdot x + 3$	7.00607	9.94147E-4
Figure 6(a), p = 200 nm	$y = \alpha \cdot x + 3$	13.86091	0.002
Figure 6(a), p = 300 nm	$y = \alpha \cdot x + 3$	20.55871	0.00297
Figure 6(a), p = 400 nm	$y = \alpha \cdot x + 3$	27.05957	0.0039
Figure 6(a), p = 500 nm	$y = \alpha \cdot x + 3$	33.38974	0.00481
Figure 6(c)	$y = \alpha \cdot x$	0.06756	0.32494
Figure 7(a), w_0 = 0.5 nm	$y = \alpha \cdot x + 0.5$	32.69383	0.00471
Figure 7(a), w_0 = 3 nm	$y = \alpha \cdot x + 3$	33.38974	0.00481
